# Comparison of microbial abundance and diversity in uterine and peritoneal fluid in infertile patients with or without endometriosis

**DOI:** 10.1186/s12905-024-02985-5

**Published:** 2024-02-29

**Authors:** Jue Zhu, Yichen Chen, Huan Chen, Yuhui Sun, Lifeng Yan, Miaohua Zhu, Liang chen, Qiming Wang, Jing Zhang

**Affiliations:** 1 Department of Gynecology, Ningbo Women and Children’s Hospital, #339 Liuting Road, Ningbo, Zhejiang China; 2 Department of Basic Research Laboratory, Ningbo Women and Children’s Hospital, Ningbo, Zhejiang China; 3 Department of Medicine, Ningbo University, Zhejiang, China

**Keywords:** Endometriosis-related infertility, Tubal obstruction-related infertility, 16S rRNA gene sequencing, Microbial community composition, Microbial differences

## Abstract

**Introduction:**

Endometriosis (EM) is a multifactorial disease that affects 10 − 15% of women of reproductive age. Additionally, 30–50% of women with EM suffer from infertility. The mechanism of infertility caused by EM has not yet been consistently explained. In recent years, studies have shown a link between infertility associated with EM and changes in the reproductive tract microbiota.

**Methods:**

In this study, we involved 26 EM patients (8 cases of stage I-II and 18 cases of stage III-IV) and 31 control subjects who were tubal obstruction-related infertility (TORI). The samples from peritoneal fluid (PF) and uterine fluid (UF) were collected and sequenced by 16 S rRNA amplicon.

**Results:**

In the comparison of microbial diversity, we found no significant differences in the microbial diversity of PF and UF between patients with stage I-II EM and those with TORI. However, there was a significant difference in microbial diversity among patients with stage III-IV EM compared to the previous two groups. *Lactobacillus* decreased in PF of EM compared to the control group, while it increased in UF. In PF, the abundance of *Pseudomonas*, *Enterococcus*, *Dubosiella* and *Klebsiella* was significantly higher in patients with stage III-IV compared to TORI patients. And in UF, the main differences existed between stage I-II EM compared to the other two groups. The abundance of *pontibacter, aquabacterium, Rikenellaceae* and so on at the genus level was significantly enriched in the EM patients with stage I-II. In the analysis based on KEGG database, EM may affect the receptivity related pathways of the endometrium by influencing changes in the uterine microbiota.

**Conclusion:**

Our results indicated that as EM progresses, the microorganisms in UF and PF keep changing. These changes in the microbiota, as well as the resulting alternations in gene functional classification, may play an important role in the infertility associated with EM.

## Introduction

Endometriosis (EM) is an estrogen-dependent condition in which cells similar to those lining the uterus grow outside of it. It causes painful periods, infertility, and pelvic masses, affecting 10–15% of women in their reproductive years [1]. However, the prevalence of the disease seems to be significantly higher in infertile women, ranging from 20 to 50% [2–4]. Women under the age of 35 with EM are at twice the risk of infertility [5]. Although the link between EM and infertility is well established, the exact mechanisms remain incompletely understood. Suspected causes include pelvic adhesions and distortions, which can block the release and transport of eggs or sperm [6]. EM may also form ovarian cysts, decrease ovarian reserve, and affect egg quality due to harmful substances in cyst fluid that can damage surrounding ovarian tissue, leading to faulty egg development and lower pregnancy chances [6]. Additional complications may involve ovulation issues and diminished endometrial receptivity [7].

Recent studies indicate a link between the imbalance of reproductive tract microbiota and EM [8–13], with EM patients showing higher levels of harmful bacteria like Gardnerella in the vagina and cervix and decreased levels of beneficial Lactobacillus [11, 12]. This dysbiosis extends to the gut and upper reproductive tract, possibly leading to chronic inflammation [11] and intestinal complications [13]. Recent research is uncovering a link between infertility and the mix of microbes in the reproductive system [14–18]. Studies have found that infertile women tend to have more asymptomatic vaginal infections [15] and a different mix of uterine bacteria [16], including those often linked to conditions like bacterial vaginosis, chronic endometritis, and endometrial polyps [16]. Fertility dysfunction may be associated with the degradation of immune tolerance due to decreased Treg cells’ function and quantity [19]. Microbial infections could further weaken the inhibitory ability of maternal Treg cells, causing placental inflammation and leading to abortion [19]. Also, imbalances in genital tract bacteria and their metabolites can impact plasma metabolite levels, possibly triggering reproductive disorders [20, 21]. Therefore, it can be seen that female infertility is closely related to the abnormality of the reproductive tract microbiota, but current research mostly focuses on the lower reproductive tract, such as the vagina and cervix.

In brief, the occurrence and development of EM is related to changes in the reproductive tract microbiota, and changes in the reproductive tract microbiota are correlated with infertility. Therefore, we speculate that the infertility caused by EM is related to changes in the reproductive tract microbiota. In the study of endometriosis-related infertility (ERI), selecting an appropriate control group is essential to gain a comprehensive understanding of this condition’s distinct physiological and microbiological features. Tubal obstruction-related infertility (TORI), with its well-defined pathological mechanism of physical blockage impeding ovum transport, serves as an optimal counterpoint in comparative studies. Unlike the pathological underpinnings of ERI, TORI provides a contrasting infertility paradigm that excludes the complication of ectopic endometrial tissues. This juxtaposition allows us to discern not only the innate repercussions of endometriosis on fertility but also the consequential shifts within the reproductive tract’s microbiota attributable to the presence of ectopic endometrial tissue. Currently, there is still no relevant research on whether there is a difference between ERI and TORI. Therefore, we conducted this study to explore the differences in bacterial communities between ERI and TORI patients.

## Materials and methods

### Patients and sampling

This study was approved by ethics committee of Ningbo Women & Children’s Hospital (EC2023-008). There were a total of 57 cases, and intraoperative samples of uterine cavity fluid and abdominal fluid were collected from the patients. According to the Revised American Society for Reproductive Medicine (rASRM) system, EM is clinically classified to 4 stages. Stage I refers to minimal disease with isolated implants and no significant adhesions. Stage II signifies mild disease with superficial implants and limited adhesions. Stage III constitutes moderate disease, characterized by multiple implants, both superficial and deep, and clear adhesions. Finally, Stage IV, the most severe, includes extensive deep implants, thick adhesions, and notable involvement of the ovaries. Patients with Stage I-II endometriosis are considered to have mild EM, while those in Stages III-IV are categorized as having moderate to severe EM. Consequently, we categorized patients with EM into two groups: the mild group, which includes Stages I-II, and the moderate to severe group, encompassing Stages III-IV. There were 26 ERI patients (8 cases of stage I-II and 18 cases of stage III-IV) and 31 TORI patients. Written informed consent was obtained from the patients to utilize their samples. Inclusion criteria: (a) Meet the diagnostic criteria for infertility; (b) Patients of reproductive age, between 18 and 45 years old; (c) Patients with EM confirmed by intraoperative macroscopic examination or postoperative pathological diagnosis, and patients of TORI group were confirmed to have only tubal obstruction during surgery. If the patients had the following conditions, they needed to be excluded: (a) History of taking antibiotics, probiotics, and hormone medications in the past 8 weeks, (b) Infertility caused by other factors, such as polycystic ovary syndrome, uterine adhesions, endometrial lesions, male factors and so on, (c) Patients with vaginitis, cervical HPV infection, and abnormal cervical TCT screening results, (d) Patients with comorbidities such as hypertension, diabetes, gastrointestinal diseases, and systemic immune system diseases. For the upper reproductive tract samples, uterine fluid (UF) and peritoneal fluid (PF) were taken during the operation. For PF samples, a syringe was used to connect the aspirator, and approximately 5 to 10 milliliters of PF were aspirated from the Douglas pouch. Immediately transfer the liquid to a sterile 15 ml centrifuge tube for subsequent processing. In the case of UF acquisition, a hysteroscope outfitted with a sterile saline infusion system was utilized. Sterile saline was carefully infused into the uterine cavity, and after allowing the solution to interact with the endometrium for a period of one minute, it was then evacuated through the hysteroscope’s outflow channel. A volume of 10 milliliters of this uterine lavage was collected and transferred into a 15 ml centrifuge tube, ensuring minimal contamination and preservation of the sample integrity. All specimens were collected consecutively from July 2022 to June 2023 and stored at -80 ℃ until DNA was extracted.

### DNA extraction and PCR amplification

Total DNA of the sample was extracted using MagPure Soil DNA LQ Kit (Magan) and the concentration and purity were determined by NanoDrop 2000 (Thermo Fisher Scientific, USA) and lipopolysaccharide gel electrophoresis. The DNA samples were stored at -20 °C for the further requirements. The V3-V4 region of the 16s rRNA genes was successfully amplified using PCR with the universal primers 343 F and 789R, with a previous study indicating that the reverse read of this amplicon has minimal impact on species classification [22].

### Library construction and sequencing

The Amplicon quality was visualized using agarose gel electrophoresis. The PCR products were purified with AMPure XP beads (Agencourt) and amplified for another round of PCR. After being purified with the AMPure XP beads again, the final amplicon was quantified using Qubit dsDNA Assay Kit (Thermo Fisher Scientific,USA). The concentrations were then adjusted for sequencing. Sequencing was performed on an Illumina NovaSeq 6000 with 250 bp paired-end reads. (Illumina Inc., San Diego, CA; OE Biotech Company; Shanghai, China).

### Bioinformatic analysis

QIIME2 software was used for alpha and beta diversity analysis. The microbial diversity in samples was estimated using the alpha diversity that includes Chao1 index and Shannon index. The unweighted Unifrac distance matrix performed by R package was used for unweighted Unifrac Principal coordinates analysis (PCoA) to estimate the beta diversity. Then the R package was used to analyze the significant differences between different groups using ANOVA statistical test. All data were shown as mean ± SD, and P-values < 0.05 were considered statistically significant.

## Results

### Basic characteristics of the study participants

A total of 26 ERI and 31 TORI patients were enrolled in this study. According to the r-AFS score, 8 patients were diagnosed with stage I -II EM and 18 patients with stage III- IV EM. In the analysis of microbial abundance and diversity, we divided the samples into three groups: stage III-IV EM (group 1), stage I-II EM (group 2), and TORI group (group 3). The basic characteristics of the patients in each group are shown in Table 1. Mean ages were 31.06 years in group 1, 28.75 years in group 2, and 30.97 years in group 3. There were no significant differences in basic demographic and clinical characteristics including age, infertility category, infertility time, endometrial thickness and visual analogue scale (VAS) between the groups. But the body mass index (BMI) was higher in group 3 and CA125 was higher in group 1.

**Table 1:** The basic characteristics of the patients

	Group 1 (*n* = 18)	Group 2 (*n* = 8)	Group 3 (*n* = 31)	P value
Age (years)	31.06 ± 5.55	28.75 ± 4.432	30.97 ± 5.295	0.538
BMI (kg/m^2^)	20.60 ± 2.83	20.88 ± 2.05	23.14 ± 2.98	0.007*
Infertility category				0.965
Primary infertility	8	4	14	
Secondary infertility	10	4	17	
Infertility time (years)	2.72 ± 2.35	2.00 ± 1.41	2.69 ± 2.02	0.672
Endometrial Thickness (mm)	8.67 ± 3.57	7.88 ± 1.96	7.43 ± 3.11	0.420
VAS scores	2.22 ± 2.56	1.13 ± 2.17	0.87 ± 1.84	0.103
CA 125 (U/ml)	106.64 ± 139.91	26.30 ± 17.63	15.35 ± 12.30	0.001*

BMI: body mass index, VAS: visual analogue scale

*P < 0.05

### Comparison of microbial abundance and diversity in PF

**Fig. 1:**
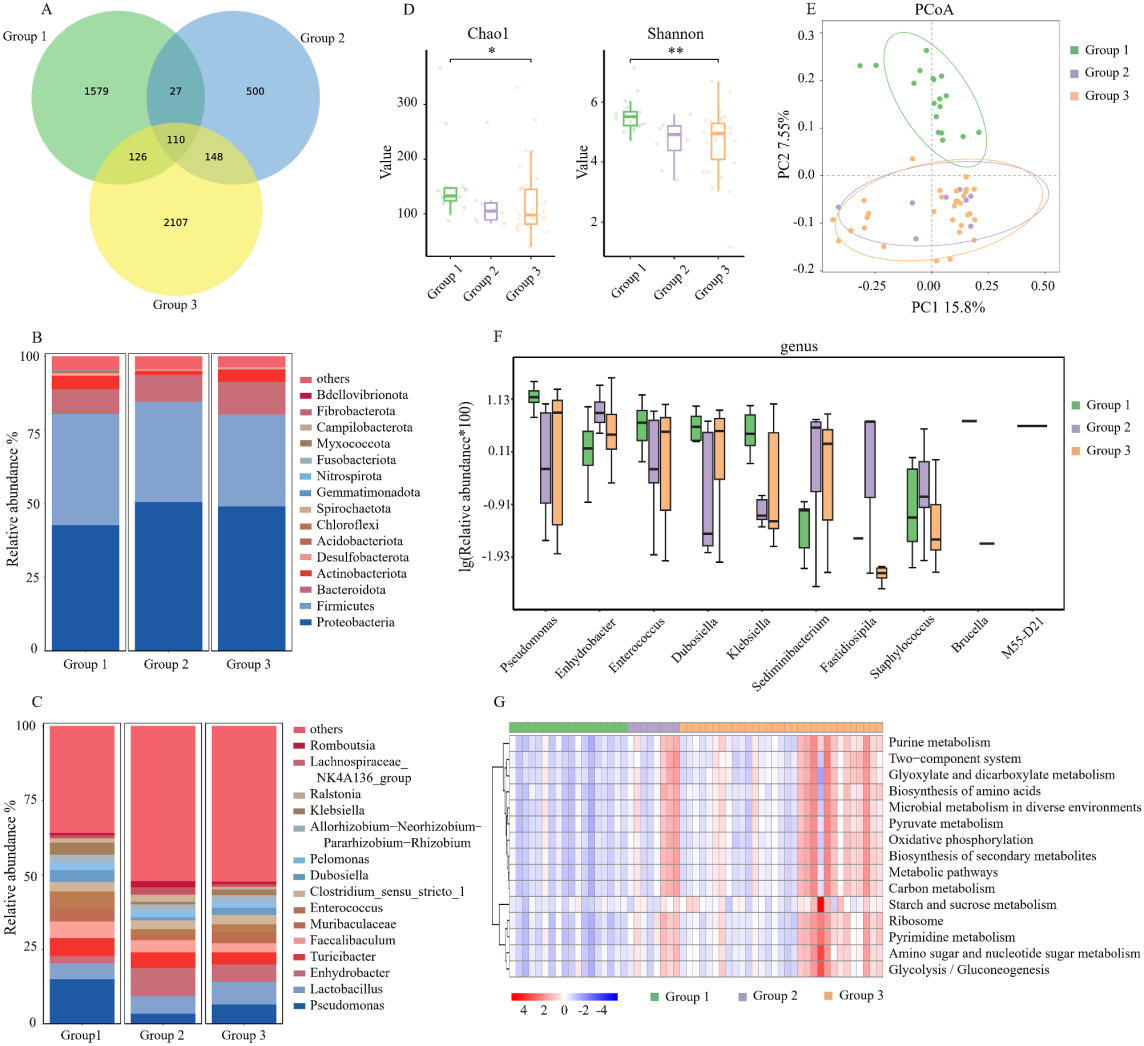
Comparison of Microbial Abundance and Diversity in peritoneal fluid. **(A**). Venn diagram. (**B**). The barplot of phylum community structure in the three groups (group 1: stage III ~ IV EMs, group 2: stage I ~ II EMs, group 3: TORI). (**C**) The barplot of genus community structure in the three groups. (**D**) The Chao1 and Shannon indices. (**E**) The principal co-ordinates analysis (PCoA). (**F**) Relative abundance in top-10 species in the three groups was indicated by ANOVA test at the genus level. (**G**) The gene functional classifications of the top 15 assembled genes in three comparative groups were demonstrated using the KEGG database. EMs: endometriosis, TORI: tubal obstruction-related infertility. ** *p* < 0.01; * *p* < 0.05

A total of 57 samples of PF were collected (18 cases of group 1, 8 cases of group 2 and 31 cases of group 3). Additionally, 55 cases of UF samples were collected (18 cases of group 1, 8 cases of group 2 and 29 cases of group 3). There were 55 paired PF and UF samples. As a result, 112 samples were subjected to the 16 S rRNA gene amplicon sequencing. Based on pyrosequencing of barcoded 16 S rRNA genes (V4-V5), we evaluated 57 PF samples and acquired 3,831,184 qualified sequences (median = 67,782) and 4597 Amplicon Sequence Variants (ASVs). A Venn diagram was used to identify the 110 out of 4597 ASVs shared by the three groups (Fig. 1A). *Proteobacteria* and *Firmicutes* were the dominant phyla in the abdominal cavity, together accounting for more than 75% of the phyla in the PF (Fig. 1B). Figure 1C depicted the top 15 bacterial community compositions at the genus level. The main genera of bacteria include *Pseudomonas, Lactobacillus, Enhydrobacter*, and *Turicibacter*. Compared to the other two groups, the abundance of *Pseudomonas* and *Turicibacter* experienced an upsurge, while the abundance of *Lactobacillus* and *Enhydrobacter* underwent a decline in group 1. The Chao1 and Shannon indices, which describe the richness and diversity of the microbiota, were significantly different among the three groups (Fig. 1D). The results revealed that the alpha diversity differences were mainly observed between Group 1 and Group 3, while no significant differences were found between Group 2 and Group 3. The microbial community structure of Group 1 was different from group 2 and group 3, according to principal co-ordinates analysis (PCoA) based on variance decomposition to represent the variations in composition (Fig. 1E).

Then, we performed ANOVA tests to analyze the differences in PF in the three groups at the genus level. Additionally, the top 10 differences in the bacterial community were shown in Fig. 1F. The abundance of *Pseudomonas*, *Enterococcus*, *Dubosiella* and *Klebsiella* was significantly higher in group 1 compared to the other two groups. Further, based on the KEGG database, the differences in gene functional classifications of assembled unigenes were shown in Fig. 1G. The pathways such as purine metabolism, two-component system, glyoxylate and dicarboxylate metabolism, biosynthesis and amino acids, microbial metabolism in diverse environments and so on were significantly different in these groups (*P* < 0.01).

### Comparison of microbial abundance and diversity in UF

**Fig. 2:**
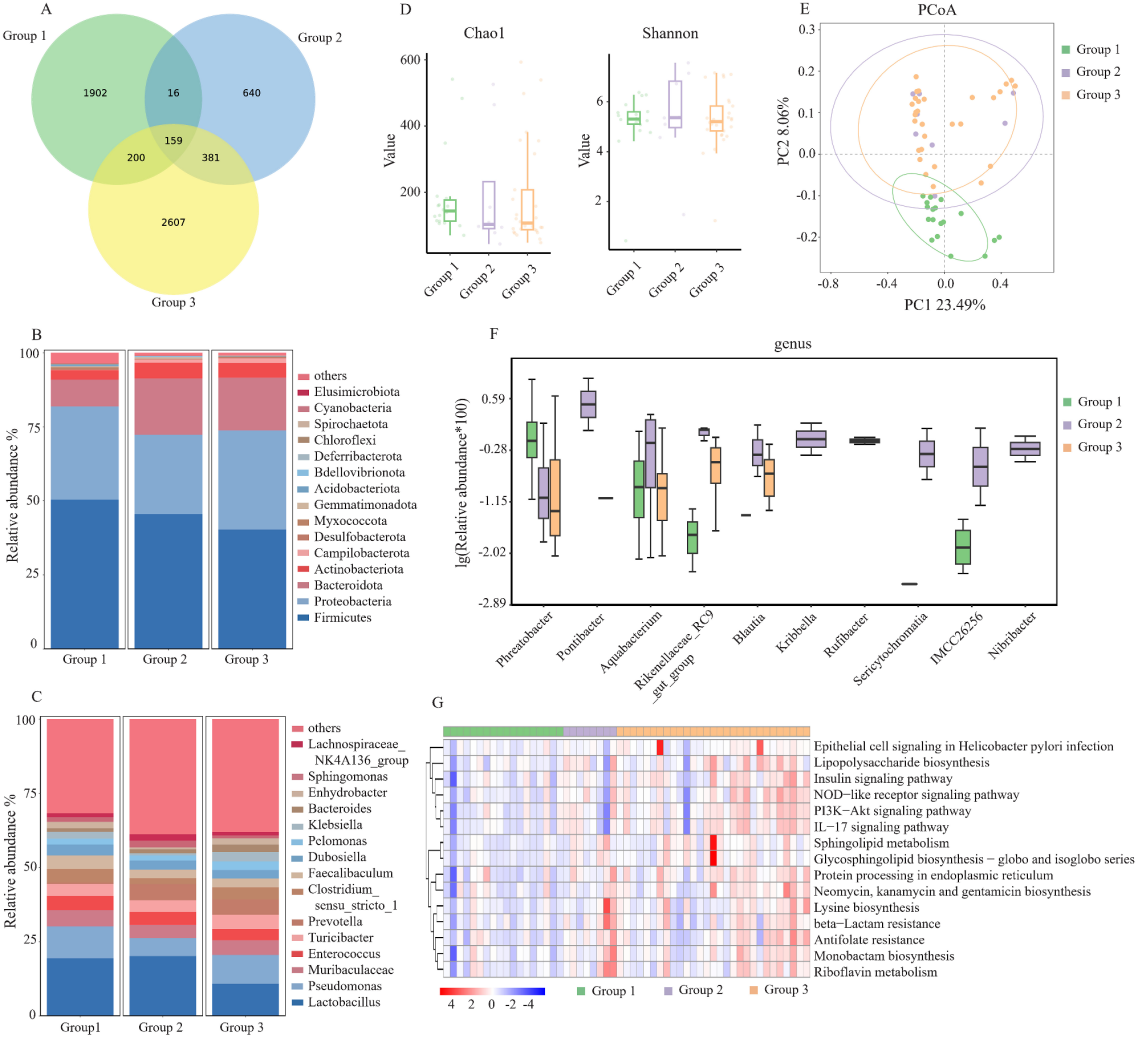
Comparison of Microbial Abundance and Diversity in uterine fluid. (**A**) Venn diagram. (**B**) The barplot of phylum community structure in the three groups (group 1: stage III ~ IV EMs, group 2: stage I ~ II EMs, group 3: TORI). (**C**) The barplot of genus community structure in the three groups. (**D**) The Chao1 and Shannon indices. (**E**) The principal co-ordinates analysis (PCoA). (**F**) Relative abundance in top-10 species in the three groups was indicated by ANOVA test at the genus level. (**G**) The gene functional classifications of the top 15 assembled genes in three comparative groups were demonstrated using the KEGG database. EMs: endometriosis, TORI: tubal obstruction-related infertility. ** *p* < 0.01; * *p* < 0.05

In the analysis of microbial community structure in UF, we also divided the samples into three groups: stage III-IV EM (group 1), stage I-II EM (group 2), and TORI group (group 3). 55 samples were evaluated. And we acquired 198,553 qualified sequences (median = 66,167) and 5905 ASVs. A Venn diagram was used to identify the 159 out of 5905 ASVs shared by the three groups (Fig. 2A). The dominant phyla were *Firmicutes*, *Proteobacteria* and *Bacteroidota* (Fig. 2B). Figure 2C depicted the top 15 bacterial community compositions at the genus level. Different from PF, the genus of intracavitary bacteria had the highest abundance of *Lactobacillus*. The proportion of *lactobacilli* was significantly higher in EM patients (19.9%) than TORI patients (10.9%) (Fig. 2C). However, in the analysis of UF microorganisms, there was no significant difference in alpha diversity among the three groups (Fig. 2D). But in the analysis of PCoA, the microbial community structure of Group 1 was different from the other two groups (Fig. 2E). This result was similar to the comparison of microbial community structures in PF.

Then, we conducted a comparison of microbial community structures in the three groups. The top 10 differences in the bacterial community were shown in Fig. 2F. The abundance of *Phreatobacter* was higher in group 1. The abundance of *Pontibacter, Aquabacterium, Rikenellaceae, Blautia, Kribbella, Rufibacter, Sericytochromatia, IMCC26256* and *Nibribacter* at the genus level was significantly enriched in the EM patients with stage I-II (*P* < 0.05). Further, based on the KEGG database, the differences in gene functional classifications of assembled unigenes were shown in Fig. 2G. Only the top 15 differences in gene function are shown in the figure. Pathways such as NOD-like receptor signaling pathway, IL-17 signaling pathway, PI3K − Akt signaling pathway, Insulin signaling pathway and so on were different in the three groups (*P* < 0.05) (Fig. 2G).

### The abundance of *Pseudomonas* and *lactobacillus* in EM patients of different stages and in patients with different types of infertility

**Fig. 3:**
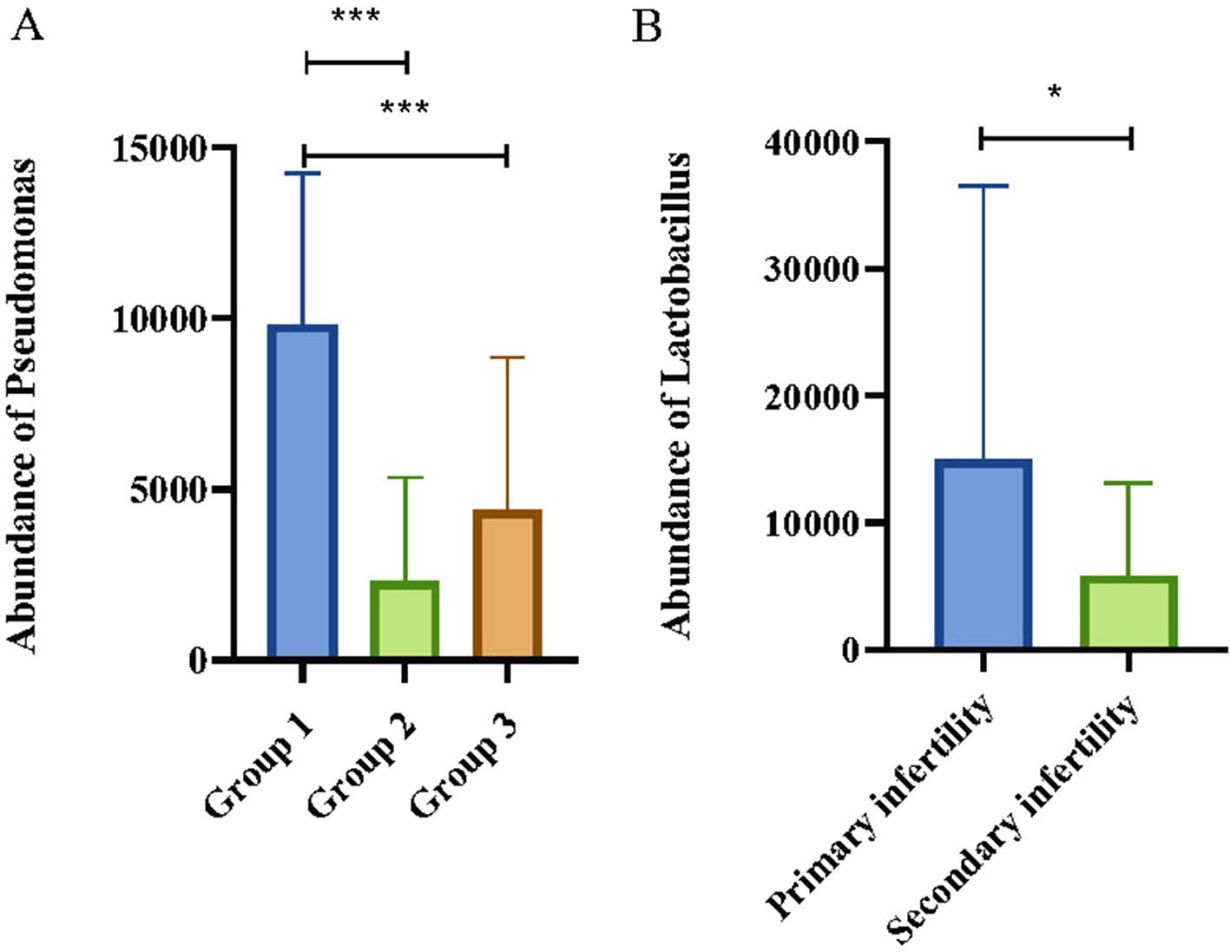
Abundance of *Pseudomonas* and *Lactobacillus*. (**A**) The abundance of *Pseudomonas* in the three groups in PF (group 1: stage III ~ IV EMs, group 2: stage I ~ II EMs, group 3: TORI). (**B**) The abundance of *Lactobacillus* in primary and secondary infertility patients in UF. PF: Peritoneal fluid; UF: Uterine fluid. *** *p* < 0.001; * *p* < 0.05

We assessed the relationship between the variations in *Pseudomonas* and *Lactobacillus* abundances and the clinical stages of EM as well as associated infertility. A closer examination of the genus in PF revealed that patients suffering from moderate to severe EM exhibit a significant increase in *Pseudomonas* abundance (Fig. 3A). In contrast, *Lactobacillus* levels did not differ markedly. Furthermore, no significant correlation was discerned between the stages of endometriosis and the abundance of either *Pseudomonas* or *Lactobacillus* in the UF. Subsequent analyses investigating the link between changes in the predominant microbial communities within UF and PF and the categories of infertility identified that *Lactobacillus* concentrations were comparatively elevated in the uterine cavities of patients with primary infertility compared to those with secondary infertility (Fig. 3B).

## Discussion

Recent research has linked the progression of EM to changes in the reproductive tract’s microbiota. However, few studies focus on the microbiome in patients with ERI. In this study, we used 16 S rRNA sequencing to profile the microbiome of the PF and UF in patients with ERI and TORI.

Our research found that there was a significant correlation between changes in the microbial communities of the UF and infertility. The results showed that *Lactobacillus*, *Pseudomonas*, and *Muribaculaceae* were key genera in UF. Other research, including Franasiak et al. [23], also noted *Lactobacillus*’s dominance in endometrial samples, and Moreno et al. linked high *Lactobacillus* abundance in endometrial fluid of fertile women (over 90%) with better pregnancy outcomes [24]. Neither patient with EM nor TORI in our study had such high *Lactobacillus* levels. According to these findings, we speculate that the decreased abundance of *Lactobacillus* in the UF may be associated with adverse reproductive outcomes. Further investigation has revealed a markedly increased level of *Lactobacillus* in UF of individuals with primary infertility when compared to those with secondary infertility. Hence, we surmise that a history of parturition may also influence the dynamics of the microbial community within the uterine environment. Therefore, in subsequent studies, further separate analyses should be conducted for patients with and without a history of childbirth.

Considering the link between UF microbiota and pregnancy outcomes, our analysis revealed notable differences in UF microbiota between early-stage EM (I-II) and other groups. We observed an increase in *Bacteroidetes*, including *Rikenellaceae*, *Blautia*, and *Rufibacter*. This aligns with previous studies indicating a rise in various bacteria like *Proteobacteria*, *Bacteroidetes*, and *Actinobacteria* in EM patients versus healthy individuals [25]. Moreover, bacteria like *Enterobacteriaceae*, *Streptococcus*, *Staphylococcus*, *Escherichia coli*, and Gram-negative bacteria, commonly found in the cervix and vagina, have been linked to lower implantation success and adverse pregnancy outcomes [24, 26–30]. *Phreatobacter, Pontibacter, Aquabacterium, and Rikenellaceae*, which were higher in UF of EM patients, were all Gram-negative bacteria. However, we must acknowledge that the smaller sample size and the marked individual variation, especially in the UF of stage I-II EM patients, may have introduced selection bias and reduced the generalizability of our findings.

To delve deeper into the effects of uterine microbiota alterations on pregnancy outcomes, we extended our analysis using the KEGG database. This analysis uncovered significant functional differences in genes within the UF, especially in pathways associated with endometritis and endometrial receptivity. Patients with TORI showed higher levels of the IL-17 signaling pathway, often increased in chronic endometritis [31]. The Insulin signaling pathway, NOD-like receptor signaling pathway, and PI3K-Akt signaling pathway were all downregulated in patients with stage III-IV EM. NOD-like receptor Pyrins-3 (NLRP3) upregulation can help in endometrial conditioning for embryo implantation [32, 33], while Insulin signaling is vital for decidualization, a key pregnancy process [34]. Our data suggest that EM might impact fertility by altering uterine microbiota, potentially affecting receptivity.

In PF, *Pseudomonas* and *Lactobacillus* were the main dominant genera. Our results align with the study conducted by Chen et al. [35], where *Pseudomonas* was found to be the most abundant microbe in PF, accounting for 13.5%. We found that an increase in the abundance of *Pseudomonas* is an important marker in patients with moderate to severe EM. The content of *lactobacilli* in PF of TORI patients accounted for 7.6%. In comparison, it accounted for only 5.7% in patients with EM, indicating a significant decrease. Furthermore, the abundance of *lactobacilli* in PF was similar between patients with EM in stages I and II (5.9%) and those in stages III and IV (5.4%). These findings were consistent with the results of Wei et al. [8]. Thus, we speculate that the decreased abundance of *Lactobacillus* in the PF may be an important marker for EMs.

In the analysis of microbial abundance differences between patients with stage III-IV and those with TORI. In PF, the abundance of *Pseudomonas*, *Enterococcus*, *Dubosiella* and *Klebsiella* were significantly higher in patients with stage III-IV compared to TORI patients. *Enterococcus* is commonly found in the intestines of humans. Previous studies have also shown that the altered composition of the intestinal microbiota induced by EM results in the translocation and infiltration of a significant number of Gram-negative bacteria outside the intestinal cavity. This leads to the destruction of intestinal tight junctions and a decrease in the expression of tight junction protein 2 [36], causing a substantial infiltration of Gram-negative bacteria outside the intestine [37]. These results suggested that in the early stages of EM, there were only minimal changes observed in the microbiota within the uterine and abdominal cavity. However, as EM progresses, the composition of the microbial community also underwent continued alterations. Our study points to a need for longitudinal studies to verify these implications and investigate causality in microbial shifts and infertility associated with EM.

## Conclusion

In summary, the implications of infertility related to EM extend beyond pelvic adhesions, anatomical distortion, ovarian dysfunction, and other direct physiological disruptions. The changes in the microbiota and the subsequent shift in gene functional profiles suggest a significant, yet underexplored, role in reproductive health. We observed dynamic variations in the microbiota associated with the UF and PF as EM progresses, indicating a potential microbial involvement in disease advancement. Recognizing the importance of these findings, it is crucial to discuss their broader implications for future research, clinical practice, and patient management in the field of gynecology and reproductive health. Future studies should aim to elucidate the direct impact of specific microbial alterations on fertility outcomes. Such research endeavors could lead to novel diagnostics and treatments, improving management strategies for patients suffering from infertility associated with EM.

## Data Availability

The data used in this study are publicly accessible and can be found in the China National Center for Bioinformation repository at [https://ngdc.cncb.ac.cn/gsub/, under the accession number: CRA013975].

## Abbreviations

EM: endometriosis
ERI: endometriosis-related infertility
TORI: tubal obstruction-related infertility
PF: peritoneal fluid
UF: uterine fluid
VAS: visual analogue scale
BMI: body mass index
PCoA: principal co-ordinates analysis
NLRP3: NOD-like receptor Pyrins-3
