# Correction: Comparison of microbial abundance and diversity in uterine and peritoneal fluid in infertile patients with or without endometriosis

**DOI:** 10.1186/s12905-024-03194-w

**Published:** 2024-06-14

**Authors:** Jue Zhu, Yichen Chen, Huan Chen, Yuhui Sun, Lifeng Yan, Miaohua Zhu, Liang chen, Qiming Wang, Jing Zhang

**Affiliations:** 1 Department of Gynecology, Ningbo Women and Children’s Hospital, #339 Liuting Road, Ningbo, Zhejiang China; 2 Department of Basic Research Laboratory, Ningbo Women and Children’s Hospital, Ningbo, Zhejiang China; 3 Department of Medicine, Ningbo University, Zhejiang, China

**Correction: BMC Women’s Health (2024) 24:148**

10.1186/s12905-024-02985-5.

Following publication of the original article [1], in the Funding Section, the grant number relating to Scientific research funds of Ningbo Natural Science Foundation Key Project was incorrectly given as 20221JCGY010631 and should have been 2022J239.

The original article has been corrected.
